# Crestal bone stability after flapless placement of sloped implants with immediate temporization in edentulous mandible. A prospective comparative clinical trial

**DOI:** 10.1002/cre2.352

**Published:** 2020-11-30

**Authors:** Algirdas Puisys, Viktorija Auzbikaviciute, Egle Vindasiute‐Narbute, Saulius Zukauskas, Kestutis Vaicekauskas, Dainius Razukevicus

**Affiliations:** ^1^ Vilnius Research Group, Private Practice VIC Clinic Vilnius Lithuania; ^2^ VIC Clinic Vilnius Lithuania; ^3^ Institute of Odontology, Faculty of Medicine Vilnius University Vilnius Lithuania; ^4^ Lithuania University of Health Science Kaunas Lithuania

**Keywords:** All‐on‐4, crestal bone stability, sloped implants, tilted implants

## Abstract

**Objectives:**

The purpose of this study was to evaluate crestal bone stability around sloped implants using the flapless procedure and compare it with conventional implants placed axially.

**Materials and methods:**

A total of 40 bone‐level implants with platform switching were used for this study for 10 patients (4 males and 6 females) in edentulous mandible. Twenty mesial conventional implants were placed in upright position and 20 distal 30° sloped implants tilted 30°. Bone loss was estimated using radiographic imaging after a 6‐ and a 12‐month follow‐up period. Comparison of the bone loss in the distal and mesial region at both implantation angles were conducted to understand the nature and progression of crestal bone loss.

**Results:**

Crestal bone loss around the sloped implants was 0.29 mm (SD = 0.292) on average, while around conventional implants it was 0.22 mm (SD = 0.202) after one‐year follow‐up. However, there was no significant difference in the average of crestal bone loss between two trial groups after 6 months (*p* < 0.243) and one‐year (*p* < 0.614) follow‐up. The results indicated a 100% implant survival rate after one‐year follow‐up. Additionally, three fixed prostheses needed realignment after fracturing during the follow‐up time.

**Conclusion:**

Considering the limitations of this study, it can be presumed that sloped and conventional implants with platform switching and conical connection have the same potential for minimal crestal bone loss.

## 1 INTRODUCTION

Prostheses on dental implants increases quality of life (QOL), especially for edentulous patients who prefer not to have removable dentures (Visscher et al., 2014). However, implantation of a foreign body may alter the bone metabolism resulting from strain and stress caused by the pressure during physical functions. Bone, being a living entity, with complex signaling pathways for remodeling, responds to external stimuli and may be a cause for marginal bone loss around the implants resulting from load pressure presented at the crestal bones (Albrektsson et al., 2017; Aldahlawi et al., 2018).

For minimal crestal bone loss, a definitive method of implantation remains yet to be defined. Factors such as timing of implant placement and loading, number of implants, prosthesis type – removable or fixed – may determine the extent of bone loss on edentulous patients (Vervaeke et al., 2015). According to standard Branemark protocol where implants are placed in upright position to have vertical vector loading forces, there is a requirement for long cantilevers (sometimes up to 20 mm) to place implants in the anterior region (Balshi et al., 2014; Drago, 2017; Poluha et al., 2015). The use of long cantilevers may result in mechanical overload of the prosthesis due to unequally distributed forces on the screw, prosthesis, abutment or implant, thereby reducing success and survival rates (Balshi et al., 2014; Krekmanov et al., 2000). Setting implants at a certain angle cantilevers may be reduced. Tilted implants may help to refuse large lateral and vertical bone augmentation, nerve lateralization and sinus lift with all leading risk of complications (Krekmanov et al., 2000; Liu et al., 2019). Moreover, the procedure allows longer implants where bone‐to‐implant contact is increased, providing primary stability.

Tilted implants have been used for a long time and show good survival rates in comparison to axially placed implants (Cavalli et al., 2016; Krennmair et al., 2016). The outcome, however, remains controversial, as some studies report greater crestal bone loss around tilted implants (Queridinha et al., 2016). With no recommended standard fixed implant angle, the question of stability and morbidity on a particular angle remains largely unanswered (Liu et al., 2019). Authors suggests angles for distal implants of between 30° to 45° (Taruna et al., 2014).

Another procedure that claims significant reduction of surgical intervention is the flapless method. Being a minimally invasive technique, the method has shown significant increase in demand among clinicians. The procedure has several advantages, such as less bleeding, minimal alteration of vascularization, swelling and morbidity. Flapless surgery being a less traumatic procedure with smaller chances of diverting blood supply from the supraperiosteum, the crestal bone could be preserved from resorption (Browaeys et al., 2015; Wang et al., 2017).

There are various reports that establish the equivalence of tilted implants to axial implants on crestal bone loss with an All‐on‐4 concept. A report by Ozan and Kurtulmus‐Yilmaz (2018) on stress distribution at 0°, 17°, 30°, and 45° shows a direct co‐relation of stress relief and tilt angle (Ozan & Kurtulmus‐Yilmaz, 2018). A study by Saber et al. (2015) came to a similar result when testing tilt angles of 0°, 15°, 30°, and 45° (Saber et al., 2015). However, other degrees of orientation and their effect on bone stability remain unexplored. The present study aims at finding the extent of crestal bone loss and the difference between the 30° sloped and conventional 0° implants of implantation with flapless surgery in edentulous patients using an All‐on‐4 approach.

## 2 MATERIALS AND METHODS

### 2.1 Study design

The subjects for this study were selected among the patients of the Vilnius Implantology Center Clinic, Vilnius, Lithuania. The protocol for this study was approved by the Bioethical Committee of the Lithuanian University of Health Science (No. BEC‐LSMU (R) ‐70). To be included in the study, the patients had to fulfill the following criteria: a) be male or female and at least 18 years of age, b) be in a generally healthy condition, with no medical contraindication for implant surgery, c) have an edentulous mandible with severe bone atrophy, d) need to have voluntarily signed the informed consent, e) have no bone augmentation procedures performed before and during implant placement, f) have an alveolar crest of at least 5.5 mm width and 10 mm height available. Subjects were excluded if they did not meet the inclusion criteria and additionally if they: a) were heavy smokers (more than 10 cigarettes/day), b) had systemic disease (diabetes, osteoporosis), c) primary stability after implant placement was not achieved. All patients had removable dentures in upper jaw.

**FIGURE 1:**
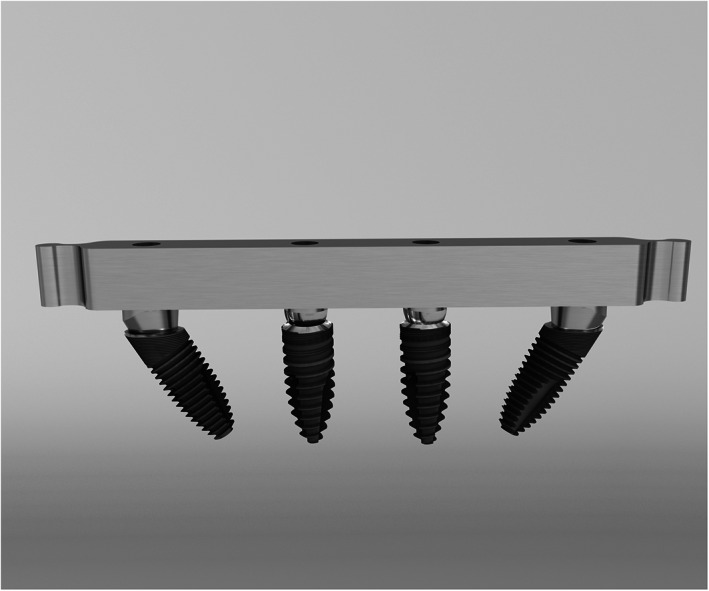
Conventional 0° mesial and 30° sloped distal implants

Initially, 10 patients (4 men and 6 women) fulfilled the inclusion criteria. Each patient received 4 implants in the mandible where 2 mesial straight implants were positioned vertically and 2 distal, 30° sloped implants were tilted 30°. Cumulatively, 40 implants were placed (Figure 1).

### 2.2 Planning

Diagnostic impressions were taken with the help of intraoral and extraoral photographs. New removable dentures were manufactured as per the anatomical features of each patient to fit the dimension and functional pattern of the patient. CBCT scans with dentures in the mouth and without were taken and used as radiological guide. Composite points of 3 mm thickness were used on dentures as a reference to match CBCT scan and dentures in implant planning software (Implant Studio, 3 shape, Denmark). Full surgical guidance was provided from the lab, including the guide for 4 implants and 3 fixation pins. Distal implants were tilted 30°.

### 2.3 Surgery

Patients received a prophylactic dose of 2 g amoxicillin (Ospamox; Biochemie, Kiel, Germany) 1 hour prior to the surgery. After administration of 40 ml, 4% articaine solution (Ubistesin; 3M ESPE, Seefeld, Germany) for local anesthesia, the surgical guide was fixed using bite registration, followed by the perforation of soft tissues using tissue punch, and the implant bed was prepared according to the manufacturer's recommendation. Four bone level implants (Medentika, Straumann Group) were placed. Two 3.5 mm implants were placed mesially and 2 sloped implants of 4.3 mm were placed distally with the flapless approach. The implants were positioned 4 mm deeper from the gingiva margin. A primary torque of >35 N/cm was achieved in all cases. Multi‐unit abutments of 4 mm height were attached and tightened at a torque of 35 N/cm. Impression and bite registration were taken, following the delivery of the relined prosthesis within 24 hours post‐surgery. Periapical X‐rays were taken using a lab‐fabricated sensor‐positioning device.

Post‐operative medication included analgesics (ibuprofen), 0.12% chlorhexidine‐digluconate (Perio‐aid; Dentaid, Spain) mouth rinse (twice a day for 7 days), and prophylactic antibiotics (amoxicillin 1,000 mg, 3 days 2 times/day) were prescribed.

Sutures were removed after 7 days, including a thorough cleaning of the prosthesis in the dental lab, and cleaning instructions were given to the patient.

After 2 months, the provisional restoration was unscrewed, and the presence/absence of bleeding was registered. The provisional restoration was relined if necessary.

Twelve months post‐operatively, a silicon impression using an individual tray and open‐tray impression transfers were taken, and screw‐retained acrylic‐based restorations were delivered.

### 2.4 Data collection and analyses

Intraoral radiographs were recorded with paralleling technique using a Rinn‐like film holder three times with each patient during the study: (i) just after placement of implant (ii) after 6 months and (iii) after 1‐year follow‐up. During the imaging process clear visibility of implants/abutments and the thread were ensured to ascertain parallelism. This was performed for both sloped and axial implants. Radiological evaluation and bone loss were estimated using RVG Windows Trophy 7.0 software (Trophy Radiologie Inc., Paris, France) with 10X magnification by an examiner. The measurement of bone loss was based on the calibration with implant diameter as reference point. Bone loss and comparison between groups and within groups was analyzed separately on distal and mesial implants. The second and the third measurement performed with 1‐month interval determined the intra‐examiner agreement. The mean difference between measurements was <0.1 mm, and the mean of three measurements was used for further analyses.

Data was analyzed using SPSS 15.0 for Windows (SPSS; Chicago, IL, USA) statistical software. A single patient was treated as a statistical unit. Mean bone loss was calculated for each group with SE. Mann–Whitney *U*‐test was applied to find differences between the groups as variables do not seem to be normally distributed. The results were considered statistically significant at *p* ≤ 0.05 with a confidence interval (CI) of 95%. The study is considered as an explorative one as no data correction of multiple testing was applied.

## 3 RESULTS

**FIGURE 2:**
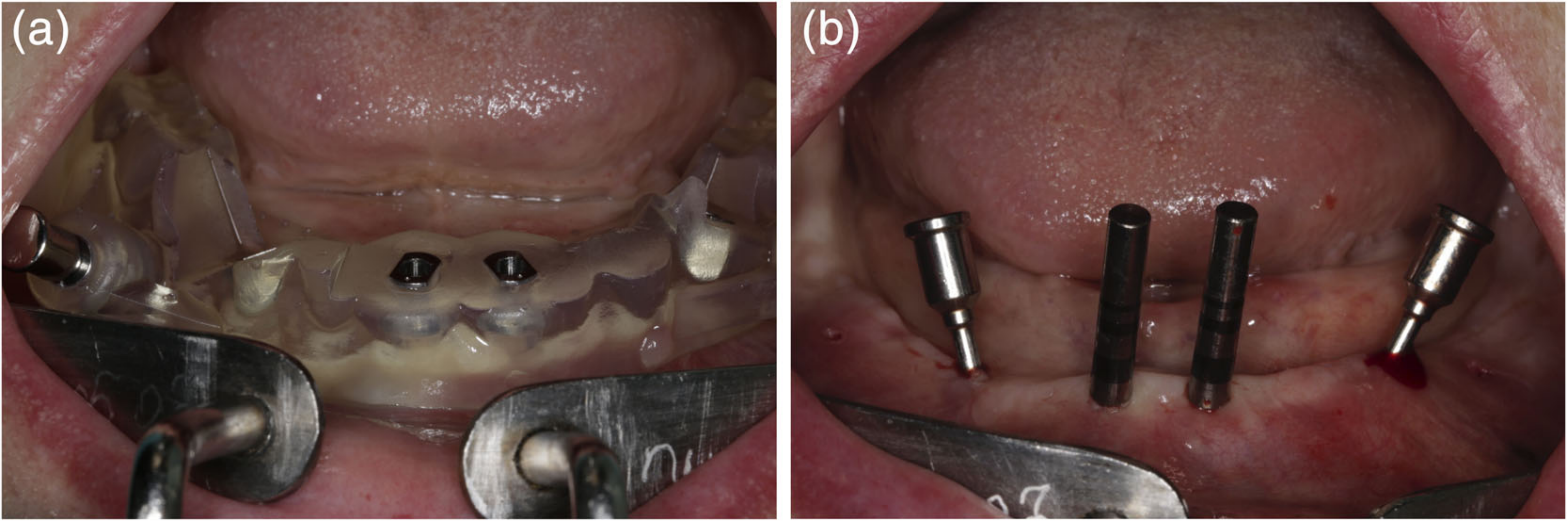
Preparation for implantation in the distal and mesial region: (a) placement of surgical guide, (b) mesial implants placed upright and distal implants tilted 30°

**FIGURE 3:**
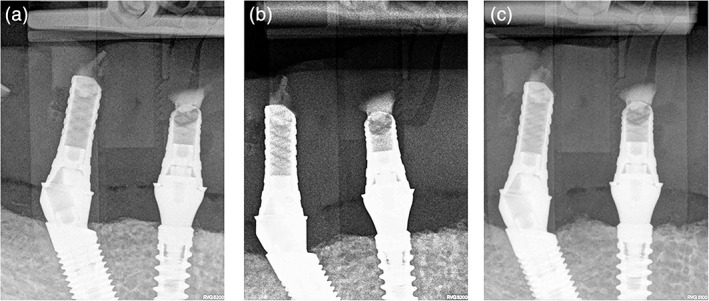
Radiographic image of axial and sloped implants indicating minimal bone loss and survival at 1‐year follow‐up: (a) just after implantation, (b) after 6 months and (c) at 1‐year follow‐up

The study included 10 patients (4 males and 6 females) with an average age of 58 (53–65) years. A total of 40 bone‐level implants with platform switching (Medentika, Straumann Group, Germany) were included in the final study. Twenty mesial implants were placed in upright position and 20 distal, sloped implants were placed tilted 30° (Figure 2). Implant survival rate was 100% after 1 year of function after flapless placement and immediate loading (Figure 3), although 3 out of 10 immediate fixed prostheses broke during follow‐up and needed to be relined. Peri‐implant probing depth was 3.76 ± 0.55 around mesial and 3.89 ± 0.43 around distal implants without any significant difference (*p* = 0.83). Bleeding index after bleeding on probing was observed 0.25 ± 0.55 and 0.3 ± 0.47 around mesial and distal implants respectively (*p* = 0.72).

After a 6‐month and 1‐year follow‐up period, mesial and distal crestal bone loss was compared between the two implant angles. No statistically significant difference was observed in bone loss in the mesial region 6 months after implantation, with bone loss of 0.16 ± 0.228 mm for conventional 0° implants compared to 30° sloped implants (0.28 ± 0.311 mm), *p* > 0.05. Similar observation was made for distal bone loss: 0.12 ± 0.16 mm and 0.23 ± 0.29 mm for 0° and 35° implants, *p* > 0.05. A 1‐year follow‐up for assessment of stability indicated no statistically significant difference between mesial and distal bone loss with axial or sloped implants, indicating equivalent performance of the two implant macro designs (Table 1). However, the difference in average bone loss between both implantation methods in terms of different time scale remains statistically insignificant.

**TABLE 1:** Among group comparison of crestal bone loss in the mesial and distal region of implants at 0° and 30° at 6‐ and 12‐month follow‐up

Timepoint	Surface	Crestal bone loss at implants		
		0°	30°	
		Mean (SD)	Mean (SD)	*p* value
6 months	Mesial	0.16 (0.228)	0.28 (0.311)	0.218
	Distal	0.12 (0.167)	0.23 (0.290)	0.348
	Average	0.14 (0.148)	0.25 (0.273)	0.243
1 year	Mesial	0.25 (0.286)	0.31 (0.342)	0.672
	Distal	0.20 (0.284)	0.27 (0.310)	0.434
	Average	0.22 (0.202)	0.29 (0.292)	0.614

Comparison of bone loss within groups aimed at understanding the progression of crestal bone loss 6 and 12 months after implantation. Among the 0° implants, we observed a statistically significant change of bone loss in the mesial region with 0.16 ± 0.22 mm and 0.25 ± 0.28 mm, *p* < 0.00, after 6 and 12 months, respectively, whereas there was no statistically significant change in the distal region with 0.12 ± 0.16 mm and 0.20 ± 0.28 mm, *p* > 0.05, at 6‐ and 12‐month follow‐up, respectively (Table 2). Crestal bone loss with 30° sloped implants followed a much different pattern, with no statistically significant change in bone loss at 6‐ (0.28 ± 0.31 mm) and 12‐month (0.31 ± 0.34 mm) follow‐up, *p* > 0.05. However, a weak statistical significance was observed for bone loss in the distal region of sloped implants, *p* = 0.047 (Table 2).

**TABLE 2:** Group comparison of crestal bone loss in the mesial and distal region of implants at 0° and 30° at 6‐ and 12‐month follow‐up

Group	Surface	Timepoint		
		6 months	1 year	
		Mean (SD)	Mean (SD)	*p* value
Crestal bone loss 0°	Mesial	0.16 (0.228)	0.25 (0.286)	0.002
	Distal	0.12 (0.167)	0.20 (0.284)	0.156
	Average	0.14 (0.148)	0.22 (0.202)	<0.001
Crestal bone loss 30°	Mesial	0.28 (0.311)	0.31 (0.342)	0.156
	Distal	0.23 (0.290)	0.27 (0.310)	0.047
	Average	0.25 (0.273)	0.29 (0.292)	0.015

## 4 DISCUSSION

The survival of implants with All‐on‐4 concept is well documented and accepted throughout the community of implantologist (Liu et al., 2019; Taruna et al., 2014). However, the degree of implantation of distal abutment is yet to be explored (Liu et al., 2019; Ozan & Kurtulmus‐Yilmaz, 2018; Saber et al., 2015). Moreover, crestal bone loss in the micro‐region of mesial and distal implants has not been reported so far. This study is the first report to evaluate crestal bone stability around conventional 0° and 30° sloped implants in the lower jaw with a flapless procedure.

This investigation did not reveal any major differences between the two implants even after follow‐up of 1 year. This outcome is in agreement with earlier findings where both conventional 0° and 30° sloped implants showed comparable peri‐implant tissue behavior (Browaeys et al., 2015; Visscher et al., 2014). Further, many other studies at different angles of implantation showed equivalent compliance with axial implants in human subjects (Ozan & Kurtulmus‐Yilmaz, 2018). Bone loss was less observed in the present study with a maximum of 0.6 mm at 1‐year follow‐up in comparison with earlier findings where crestal bone loss was more than 1 mm Puisys & Linkevicius, 2015 or around 0.5 mm (Visscher et al., 2014). The difference may be due to vertical soft tissue thickness, which may influence crestal bone stability (Linkevicius et al., 2009, 2015; Puisys & Linkevicius, 2015) as thickness was 4 mm in the present study. It can be speculated that different thicknesses of soft tissue may have been present in different patients, leading to a high SD value. The finding is further supported by a comparative split‐mouth study by Vervaeke et al. (2015) where increased bone loss was observed in patients with thinner soft tissues(Vervaeke et al., 2015). Further, Linkevicius et al. (2009) reported that no bone loss or not more than 0.5 mm were observed in almost 85% of the implants in thick mucosal tissue around implants with platform switching after 1 year. Whereas, almost 70% of implants in thin soft tissue showed more than 1.00 mm of bone loss at the same time (Linkevicius et al., 2009). Another study by Puisys and Linkevicius (2015) presents crestal bone loss of 1.22 mm in thin and 0.2 mm in thick tissue (Puisys & Linkevicius, 2015), supporting the theory that vertical soft tissue thickness is a major determining factors in preserving crestal bone. In the present study, all implants were placed 4 mm subgingivally, so that bone re‐modeling was not influenced by the re‐establishment of biological width.

Although both methods of implantation showed equal overall crestal bone loss, there was a statistically significant increase in the mesial region after 1 year of implantation compared to the distal region in axial or sloped implants, which might contribute significantly toward the prosthetic survival of implants. Further investigation of the underlying mechanism behind the observation may be required.

Another issue that may be discussed is the size of platform switching. It has been suggested that the degree of the implant‐abutment size mismatch in platform switching might be important for the amount of crestal bone loss (Atieh et al., 2010). Otherwise, if the implant has a small mismatch, there could be not enough space for the re‐establishment of biological width and the micro gap will be close to the bone. It was found that 0.4 mm of platform switching is suitable to preserve crestal bone if the thickness of soft tissue is 3 mm and more. Therefore, a prompt 0.4 mm of platform switching was provided on implants used in the study. Further, we have placed multiunit abutment of different height, avoiding placement acrylic below the gingiva in order to prevent formation of biofilm and peri‐implantitis.

Although this study did not have a control group that could establish the effect of flapless surgery on crestal bone loss, there are several reports on the advantages of the procedure that flap elevation of periosteum interrupts the nutrition of superficial bone. Simple elevation and the closure of periosteum without any manipulations may lead to some bone resorption around the teeth, and the flapless implant placement has the advantage to preserve crestal bone due to keeping blood vessels undamaged (Browaeys et al., 2015; Wang et al., 2017).

An interesting remark could be made regarding immediate temporaries. Removable dentures were relined and transformed into fixed dentures. Implant placement was done in the optimal condition that is, >5 mm wide bone crest and with the use of abutments avoiding the acrylic be positioned under the mucosa.

Same‐day delivery and low additional costs were advantages. However, relining dentures without the support of a metal frame leads to higher risk of mechanical complications. This was exactly what had happened in 3 out of 10 cases in 12 months. Patients were informed to show up immediately to fix the prosthesis if something like this happened and no additional complications were seen.

The present study has several limitations. The validity of the results might be limited due to the small number of patients and short observation time as well as the results in the lower jaw only. Thus, additional studies may be required to evaluate the effect of sloped implants in the maxilla. On the other hand, this study shows that sloped implants together with thick soft tissue vertically and multiunit abutment placed right after implantation may predispose stable bone.

## 5 CONCLUSION

There is no significant difference between crestal bone loss in the mesial and distal region of conventional 0° and 30° sloped implants with flapless surgery in mandible. The study needs further refinement with larger sample size and longer durations of follow‐up study on implant survival.

## CONFLICT OF INTEREST

Authors state no conflict of interest.

## AUTHOR CONTRIBUTIONS

Algirdas Puisys conceived the idea. Algirdas Puisys and Saulius Zukauskas placed the implants and followed the patients. Kestutis Vaicekauskas and Egle Vindasiute‐Narbute restored the implants. Algirdas Puisys and Viktorija Auzbikaviciute collected the data. Dainius Razukevicus and Algirdas Puisys analysed the data, drafted the paper. Algirdas Puisys led the writing.

## Data Availability

The date that support the findings of this study are openly available.
